# A modified perioperative regimen for deceased donor kidney transplantation in presensitized recipients without prior desensitization therapy

**DOI:** 10.3389/fimmu.2023.1223567

**Published:** 2023-07-05

**Authors:** Zhiliang Guo, Daqiang Zhao, Rula Sa, Lu Wang, Songxia Li, Guangyuan Zhao, Lan Zhu, Gang Chen

**Affiliations:** ^1^ Institute of Organ Transplantation, Tongji Hospital, Tongji Medical College, Huazhong University of Science and Technology, Wuhan, China; ^2^ Key Laboratory of Organ Transplantation, Ministry of Education, Chinese Academy of Medical Sciences, Wuhan, China; ^3^ NHC Key Laboratory of Organ Transplantation, Chinese Academy of Medical Sciences, Wuhan, China; ^4^ Key Laboratory of Organ Transplantation, Chinese Academy of Medical Sciences, Wuhan, China

**Keywords:** kidney transplantation, presensitization, acute rejection, antibody-mediated rejection, deceased donor

## Abstract

**Background:**

Renal transplantation in HLA-presensitized recipients entails an increased risk of antibody-mediated rejection (AMR) and graft loss. There is currently no accepted standard treatment protocol that can help transplant surgeons safely perform deceased donor (DD) kidney transplantation in presensitized patients without pretransplant desensitization.

**Methods:**

Fifty-one panel-reactive antibody (PRA)-positive recipients and 62 PRA-negative retransplant recipients (control) who received DD renal transplantation were included. Patients in the presensitized group (donor-specific antibody [DSA]-positive, n=25; DSA-negative, n=26) without desensitization received a modified perioperative treatment starting on day 0 or -1 with rituximab, thymoglobulin, and low daily doses of intravenous immunoglobulin (IVIG, 10-20 g/d, for 14 days). Plasmapheresis was performed once before surgery in DSA-positive recipients.

**Results:**

The median follow-up time was 51 months in the presensitized group and 41 months in the control group. The incidence of early acute rejection (AR) and AMR (including mixed rejection) was 35.3% and 13.7% in the presensitized group, respectively, significantly higher than in the control group (14.5% and 1.6%, respectively). Within the presensitized group, the DSA-positive subgroup had more AMR than the DSA-negative subgroup (24.0% vs. 3.8%), but the incidence of T cell-mediated rejection was comparable (20.0% vs. 23.4%). In the presensitized group, all rejections were successfully reversed, and graft function remained stable during follow-up. The 1-year and 3-year survival rates of the grafts and recipients in this group were 98.0%.

**Conclusion:**

With a modified IVIG-based perioperative regimen, excellent intermediate-term graft and recipient survival outcomes can be achieved in presensitized patients who received DD kidney transplantation without prior desensitization.

## Introduction

Human leukocyte antigen (HLA) sensitization caused by previous kidney transplantation is one of the main barriers for patients with end-stage renal disease to access to retransplantation (1). In addition, pregnancy and blood transfusions can also expose a small number of patients on the waiting list to foreign HLA antigens before they receive their first-time kidney transplant, leading to HLA sensitization (2, 3). As compared to non-sensitized patients, HLA-presensitized patients have a higher risk of acute rejection (AR) after renal transplantation, and especially antibody-mediated rejection (AMR) (4–6). Therefore, they are often forced to wait for a long time because of the difficulty in obtaining suitable HLA-matching kidney donors.

In order to shorten the waiting time for HLA-presensitized patients, pre-transplant desensitization therapy may be considered. However, the overall effectiveness of current desensitization regimens, which usually include plasmapheresis (PP), intravenous immunoglobulin (IVIG), and rituximab, is limited, especially in highly sensitized patients (7–9). In addition, pre-transplant desensitization therapy is more feasible in living relative kidney transplantation because of better HLA matching and the fact that the surgery is planned, but more difficult in deceased donor (DD) kidney transplantation because the surgery and donor’s identity cannot be planned in advance (10). Therefore, an important clinical issue in the field is determining how to increase the chances of success for HLA-presensitized patients waiting for DD kidney transplantation as well as how to ensure the safety of the transplantation process.

Given the use of screening for donor-recipient HLA matching and the application of perioperative treatments, successful DD kidney transplantation can now be achieved in HLA-presensitized (including DSA-positive) patients without prior desensitization. For example, a French center has reported that DSA-positive patients can receive renal transplantation across the HLA barrier, with the use of an intensified post-transplant desensitization involving high-dose IVIG, plasma exchanges, and eventually rituximab starting on the day of surgery (5). Considering that the first 2 weeks after transplantation is the high-risk period for acute AMR, we have designed a simpler perioperative regimen. In addition to rituximab and thymoglobulin induction therapy, our regimen consisted mainly of daily administration of IVIG (10-20g) for the first 2 weeks after transplantation to prevent the rebound of preformed DSA and the generation of induced DSA resulting from an anamnestic response by memory B cells (11, 12). Under close DSA monitoring, PP/IVIG therapy was only used in patients with AMR or in patients with persistently high levels of DSA that impeded renal function recovery. Using this protocol, we performed DD kidney transplantation in 51 presensitized patients (25 of whom were DSA-positive) and achieved excellent early and intermediate-term outcomes.

## Patients and methods

### Study population

This study was a retrospective single-center study. Presensitized recipients and non-presensitized retransplant recipients who received renal transplantation from a deceased donor (DD) in our hospital between May 2015 and August 2022 were included. All recipients were adults who received ABO-compatible kidney transplantation and were negative for pre-transplant complement-dependent cytotoxicity (CDC) tests. Based on the pre-transplant results of HLA antibody detection, the patients were divided into a panel-reactive antibody (PRA)-positive presensitized group and a PRA-negative retransplant group (control). We selected PRA-negative retransplant patients as controls to better investigate the effects of pretransplant PRA or DSA on acute rejection and graft survival after transplantation. The PRA-positive group was further divided into two subgroups (DSA-positive and DSA-negative) according to the presence or absence of DSA at the time of transplantation.

All donor grafts were donated to the Red Cross Society of the province and allocated by the China Organ Transplant Response System. The study procedures were approved by the ethics committee of our hospital (TJ-IRB20221138) and performed in accordance with the national program for deceased organ donation in China (13). The clinical and research activities being reported are consistent with the principles of the declaration of Istanbul as outlined in the “Declaration of Istanbul on Organ Trafficking and Transplant Tourism.”

Data on transplants and hospitalizations as well as follow-up data were collected from hospital records. The follow-up ended on March 31, 2023. Baseline characteristics of each group and subgroup were collected and compared. The clinical outcomes between the two groups and the two subgroups were compared, including the incidence of primary non-function (PNF), delayed graft function (DGF), rejection, proteinuria, perioperative hematoma, post-transplant CMV infection, BK virus infection, EB virus infection, renal graft function, and 1-year and 3-year graft and patient survival.

### Immunosuppression protocols and strategies

**Figure 1:**
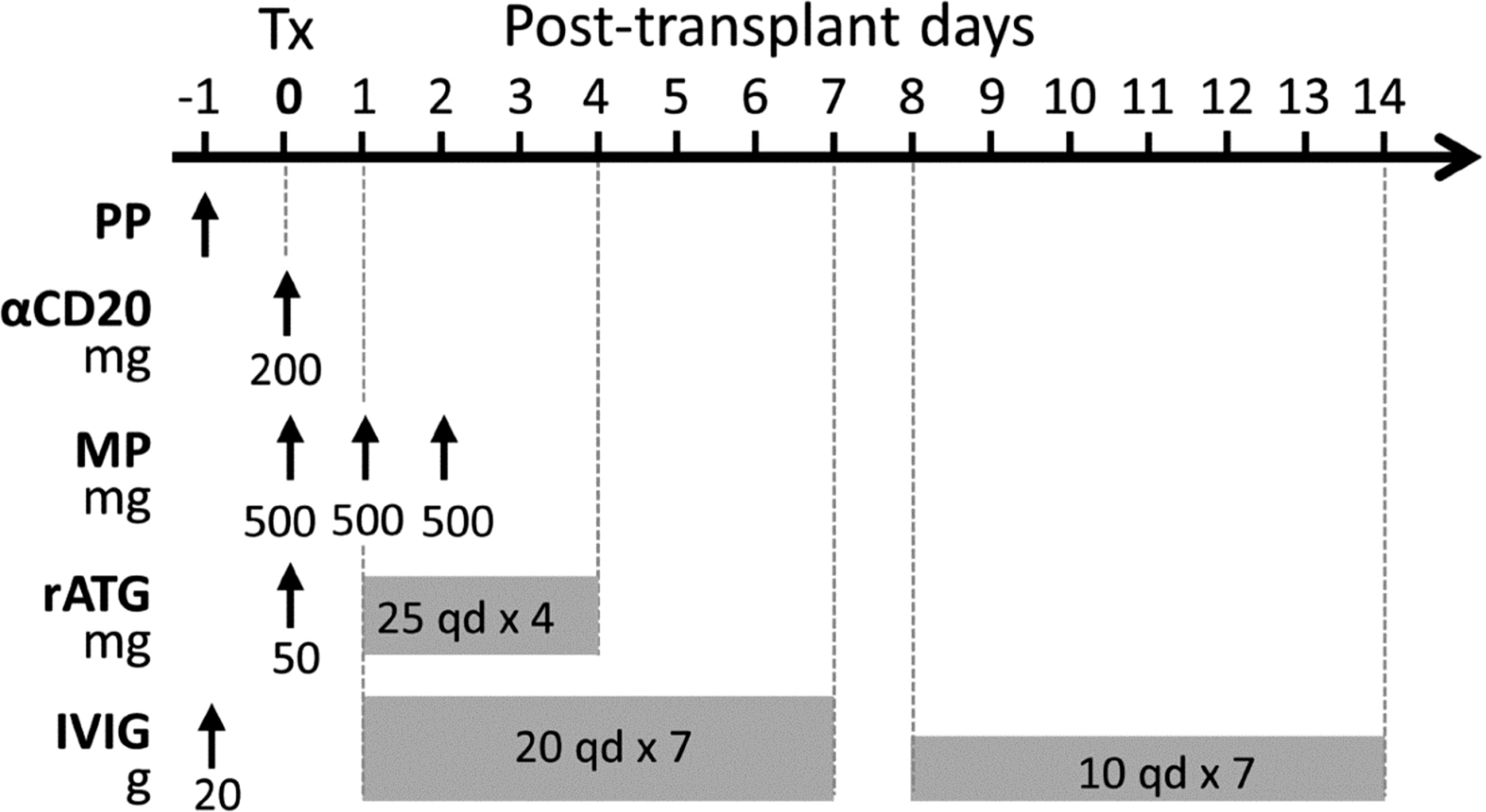
A modified perioperative treatment protocol for pfDSA-positive recipients. The treatments included 1) PP/IVIG performed once on the day before surgery; 2) preoperative low-dose rituximab (200 mg); 3) low-dose thymoglobulin induction therapy (50 mg on day 0 and 25 mg/d on days 1-4); 4) methylprednisolone (500 mg/d on day 0-2); 5) daily use of IVIG for 2 weeks (20g/d on days 1-7 and 10 g/d on days 8-14).

In our center, we have developed a modified perioperative treatment protocol for presensitized recipients in 2015, involving: (1) a single dose of preoperative rituximab (200 mg); (2) a five-day course of thymoglobulin induction therapy (50 mg on day 0 and 25 mg/d on days 1-4); (3) daily use of IVIG for 2 weeks (20 g/d on days 1-7 and 10 g/d on days 8-14); (4) an urgent use of plasmapheresis (PP)/IVIG on the day before surgery in DSA-positive recipients (**Figure 1**). In addition, DSA was monitored weekly for the first month after transplantation, and renal biopsy was considered according to the changes in DSA and the extent of the recovery of renal graft function. Additional PP/IVIG therapy was given to patients with biopsy-diagnosed AMR or patients with persistently high levels of DSA that impeded renal function recovery.

Based on the physician’s experience, PRA-negative retransplant recipients received induction therapy with either thymoglobulin (25 mg/d on days 0-2) or basiliximab (20 mg on days 0 and 4). All patients in this study received maintenance immunosuppressive therapy with tacrolimus/cyclosporine, mycophenolate mofetil and steroids. Methylprednisolone was given intravenously (500 mg/day, days 0-2), followed by oral doses of prednisone at 50 mg/day, which were then tapered every other day to a maintenance dose of 10 mg/day. Tacrolimus was started on day 3, with a targeted trough level of 7-10 ng/ml initially and 6-8 ng/ml after 3 months. Cyclosporine maintenance therapy was also considered for patients at risk for post-transplant diabetes, with an initial targeted trough level of 150-200 ng/ml and 130-180 ng/ml after 3 months. Mycophenolate mofetil was administered at a dose of 1.5-2 g/day and then reduced to 1-1.5 g/day according to the individual’s white blood cell count.

### HLA typing and HLA antibody detection

HLA typing (A, B, DR, and DQ) of donors and recipients was determined by the reverse SSO DNA typing assay (LABType SSO, OneLambda Inc. Canoga Park, CA, USA) at our center before April 2020. Since then, we have updated to a second-field typing for HLA-A, B, C, DRB1, DQA1, DQB1, DQB3/4/5, DPA1, and DPB1 on LABScan3D™ system.

Before January 2019, Flow-PRA (FlowPRA Class I & II Screening Test, OneLambda Inc. Canoga Park, CA, USA) was used to detect HLA antibodies in recipients’ sera. After January 2019, mixed-antigen bead assay was performed on a Luminex platform to detect serum HLA antibodies of recipients. HLA antibody-positive sera were further detected by single-antigen bead assay (LABScreen™ Single Antigen Beads, OneLambda Inc., Canoga Park, CA) on the Luminex platform (14). Antibodies were determined by measuring the mean fluorescence intensity (MFI) of each single-antigen bead. Adjusted raw MFI values >1,000 were defined as positive reactions. Class I and II PRA values were separately calculated at the MFI cut-off level of 1,000 (calculator: https://www.transplanttoolbox.org/nmdp_cpra/).

### CDC assays

Before transplantation, serum from all recipients was measured for CDC by the standard microlymphocytotoxicity test. For presensitized recipients, Flow-CDC assays were further performed using donor lymphocytes isolated from peripheral blood as the target cells, as described previously (15).

### Rejection diagnosis

AR was diagnosed by renal biopsy based upon the Banff 2013, 2017, or 2019 Schema, and ultimately confirmed by Banff 2019 criteria. When a tissue analysis was not available, the clinical diagnosis was based on an otherwise unexplained elevation of serum creatinine levels, coupled with appropriate physical signs (including edema, oliguria, fever, or weight gain). All allograft biopsies were routinely stained for hematoxylin and eosin (HE) and subjected to immunohistochemical staining for C4d.

### Statistical analysis

In our descriptive statistical analysis, results are expressed as numerical values, with percentages for categorical variables. Continuous variables are presented as mean values with standard deviation if normally distributed and otherwise as medians and IQRs. Baseline characteristics between groups were compared with Student’s *t*-test for normally distributed continuous variables and with the Mann-Whitney rank sum test when the data failed either the normality test or the equal variance test. For categorical covariates, *P*-values were generated by chi-squared analysis or Fisher’s exact test. Graft and patient survival were evaluated by the log-rank (Mantel-Cox) test and Kaplan-Meier method. Statistical analysis was performed using SPSS software (version 26), and GraphPad Prism software (version 9.0). *P* values <0.05 were considered statistically significant.

## Results

### Patient population

The transplants in this 113-patient cohort consisted of 51 kidney transplants into PRA-positive presensitized recipients (the presensitized group) and 62 kidney retransplants into preoperatively PRA-negative recipients (the control group). The patients in the presensitized group were further divided into a DSA-positive subgroup (n=25) and a DSA-negative subgroup (n=26) on the basis of the presence or absence of DSA at the time of transplantation.

Baseline and demographic characteristics are shown in **Table 1**. Patients in both the presensitized and control groups were predominantly male (64.7% and 72.6%, respectively). In the presensitized group, there were 10 (19.6%) primary transplant recipients who were sensitized by pregnancy or blood transfusion, and in the control group, all the patients were second and third transplant recipients (*P*<0.001). As compared to the recipients in the control group, the presensitized recipients were slightly older (mean, 47.4 vs. 42.3 y, *P*<0.05), and they had a longer time of pretransplant dialysis (median, 18 vs. 10 months, *P*<0.05). In the presensitized group, 16 patients (31.4%) were mildly sensitized (peak PRA: 10%-50%), 20 (39.2%) were moderately sensitized (PRA: 50%-80%), and 15 (29.4%) were highly sensitized (PRA ≥80%). Among these presensitized patients, 92% of the DSA-positive subgroup were moderately to highly sensitized, whereas only 46.2% of the DSA-negative subgroup showed moderate to high sensitization (*P*=0.001), indicating a higher degree of sensitization in the DSA-positive subgroup.

**Table 1:** Recipient, donor and transplant demographics.

	Presensitized PRA^+^ (n=51)	Control PRA^-^ re-Tx (n=62)	P value	Presensitized		
				DSA^+^ (n=25)	DSA^-^ (n=26)	P value
Recipient characteristics						
Male, n (%)	33 (64.7%)	45 (72.6%)	0.368	16 (64.0%)	17 (65.4%)	1
Age (y), mean ± SD	47.4 ± 10.5	42.3 ± 11.7	0.034	47.9 ± 10.5	46.8 ± 10.6	0.888
Months on dialysis, median (IQR)	18.0 (8.0, 33.0)	10.0 (6.0, 12.3)	0.021	18.0 (9.0, 36.5)	20.0 (7.3, 33.8)	0.792
N of previous Tx, N (0/1/2)	10/34/7	0/59/3	<0.001	5/16/4	5/18/3	0.818
Peak PRA, n (%)						
<10%	0	62	<0.001	0	0	–
10% - 50%	16 (31.4%)	0	<0.001	2 (8.0%)	14 (53.8%)	0.001
50% - 80%	20 (39.2%)	0	<0.001	13 (52.0%)	7 (26.9%)	0.067
>80%	15 (29.4%)	0	<0.001	10 (40.0%)	5 (19.2%)	0.104
Donor characteristics						
Age (y), mean ± SD	43.7 ± 14.1	45.4 ± 12.2	0.692	44.3 ± 10.4	43.2 ± 16.6	0.801
DBD donor, n (%)	47 (92.2%)	54 (87.1%)	0.385	23 (92.0%)	24 (92.3%)	1
DCD donor, n (%)	4 (7.8%)	8 (12.9%)	0.385	2 (8.0%)	2 (7.7%)	1
Transplant characteristics						
HLA-A/B/DR/DQ MM, mean ± SD	4.1 ± 1.8	5.4 ± 1.5	<0.001	4.0 ± 1.6	4.1 ± 2.0	0.801
Cold ischemia time (h), mean ± SD	13.3 ± 3.4	13.7 ± 3.0	0.417	13.1 ± 3.5	13.4 ± 3.3	0.720
Induction therapy, N						
Basiliximab/Thymoglobulin	0/51	10/52	0.002	0/25	0/26	–
Maintenance therapy, N						
Tac/CsA	42/9	56/6	0.214	20/5	22/4	1
Follow-up (m), median (IQR)	51 (18, 68)	41 (22, 61)	0.775	41 (16, 64)	54 (18, 78)	0.341

PRA, panel-reactive antibodies; DSA, donor-specific antibody; re-Tx, retransplantation; Tx, transplantation; DBD, donation after brain death; DCD, donation after circulatory death; MM, mismatch; SD, standard deviation; IQR, interquartile range; Tac, tacrolimus; CsA, cyclosporine A. The symbol "-", indicates that there is no comparison between the two groups of data.

Most of the donor kidneys in the two groups were from brain-dead donors, and the main causes of death were brain trauma and intracerebral hemorrhage. There were no significant differences between groups or subgroups in terms of donor age, donor category, or cold ischemic time. With respect to tissue typing, the donor-recipient HLA-A, -B, -DR, -DQ mismatch grade was slightly lower in the presensitized group than in the control group (4.1 vs. 5.4, *P*<0.001). In the presensitized group, all patients received induction therapy with thymoglobulin, whereas in the control group, 52 patients (83.9%) received thymoglobulin, and 10 patients (16.1%) received basiliximab induction therapy. There were no significant differences in maintenance immunosuppressive regimen between the groups or the subgroups.

### Early graft function and acute rejection

Of the 113 patients, only one patient in the control group had PNF of the renal graft. The incidence of DGF in the control group was relatively higher than that in the presensitized group (25.8% vs. 11.8%, *P*=0.04). The overall incidence of early AR within 3 months after transplantation in the presensitized group (35.3%) was significantly higher than that in the control group (14.5%) (*P*=0.01, **Table 2**). Further analysis showed that the incidence of antibody mediated rejection (including AMR alone and mixed rejection) in the presensitized group was significantly higher than that in the control group (13.7% vs. 1.6%, *P*<0.05). T-cell-mediated rejection (TCMR) remained the dominant type of AR in both groups (presensitized group, 21.6% vs. control group, 12.9%, *P*>0.05).

**Table 2:** Transplant outcomes.

	Presensitized PRA^+^ (n=51)	Control PRA^-^ re-Tx (n=62)	*P* value	Presensitized		
				DSA^+^ (n=25)	DSA^-^ (n=26)	*P* value
Primary non-function, n(%)	0	1 (1.6%)	1	0	0	–
Delayed graft function, n(%)	6 (11.8%)	16 (25.8%)	0.040	3 (12.0%)	3 (11.5%)	1
Acute rejection (<3m)	18 (35.3%)	9 (14.5%)	0.010	11 (44.0%)	7 (26.9%)	0.202
AMR+Mixed rejection, n(%)	2 + 5 (13.7%)	0 + 1 (1.6%)	0.022	2 + 4 (24.0%)	0 + 1 (3.8%)	0.049
TCMR, n(%)	11 (21.6%)	8 (12.9%)	0.220	5 (20.0%)	6 (23.4%)	1
*De novo* DSA, n(%)	4 (7.8%)	4 (6.5%)	1	2 (8.0%)	2 (7.7%)	1
Hematoma, n(%)	2 (3.9%)	3 (4.8%)	1	0	2 (7.7%)	0.490
Hospital stay (d), mean ± SD	33.9 ± 19.7	24.7 ± 12.4	<0.001	41.8 ± 23.4	26.3 ± 11.4	<0.001
CMV viremia, n(%)	3 (5.9%)	4 (6.5%)	0.608	3 (12.0%)	0	0.110
BK virus, n(%)	7 (13.7%)	4 (6.5%)	0.219	3 (12.0%)	4 (15.4%)	1
EB virus, n(%)	3 (5.9%)	6 (9.7%)	0.510	0	3 (11.5%)	0.235
Pneumonia, n(%)	7 (13.7%)	10 (16.1%)	0.722	5 (20.0%)	2 (7.7%)	0.248
Proteinuria, n(%)	9 (17.6%)	12 (19.4%)	0.816	4 (16.0%)	5 (19.2%)	1
1-y graft survival rate	98.0%	98.4%	0.888	96.0%	100%	0.327
3-y graft survival rate	98.0%	90.5%	0.171	96.0%	100%	0.327
1-y patient survival rate	98.0%	98.4%	0.888	96.0%	100%	0.327
3-y patient survival rate	98.0%	98.4%	0.888	96.0%	100%	0.327

PRA, panel-reactive antibodies; DSA, donor-specific antibody; re-Tx, retransplantation; AMR, antibody-mediated rejection; TCMR, T cell-mediated rejection; CMV, cytomegalovirus. The symbol "-", indicates that there is no comparison between the two groups of data.

**Figure 2:**
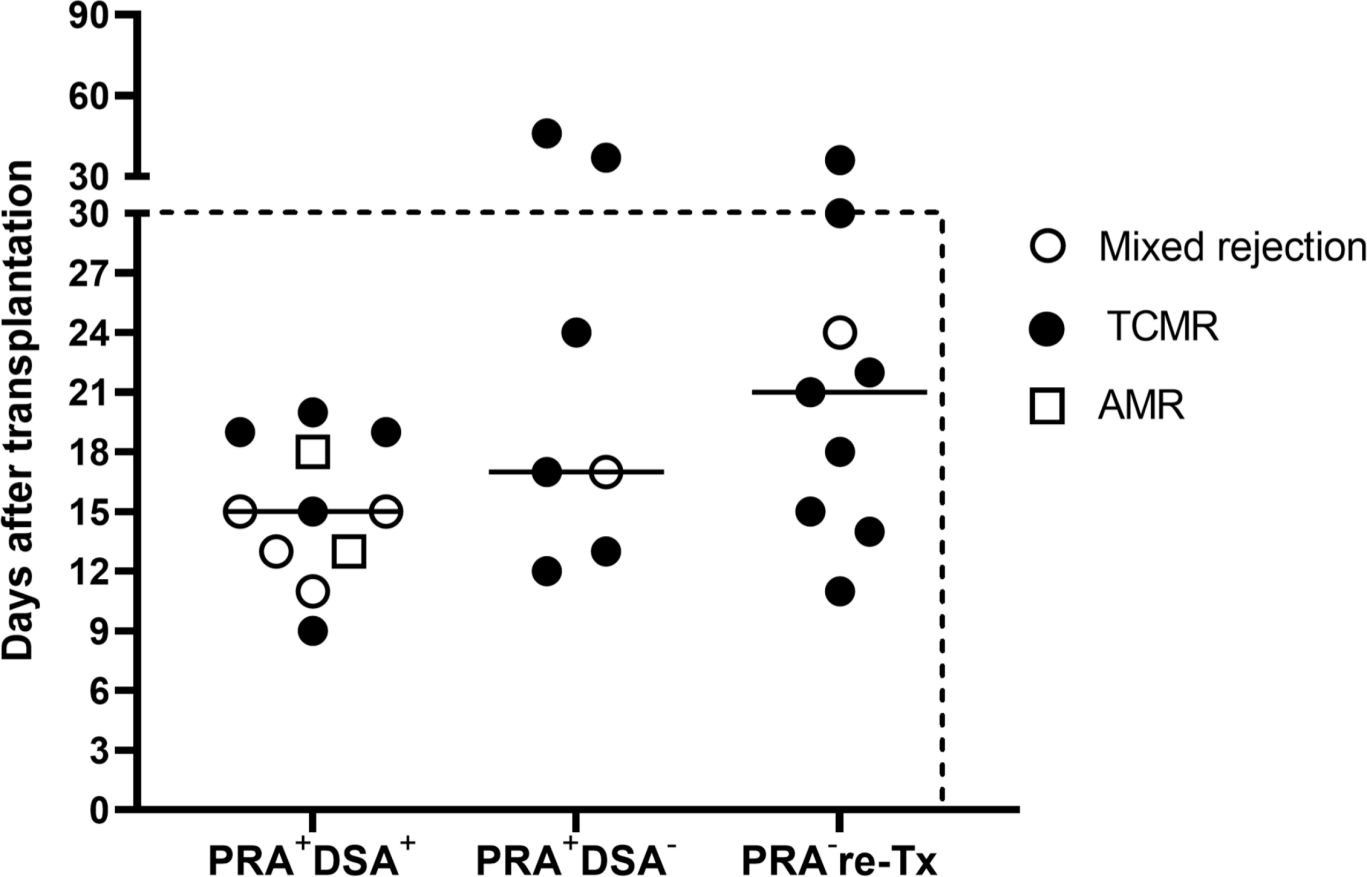
Time of occurrence and types of all instances of acute rejection. The majority (88.9%) of the acute rejection (AMR, mixed rejection, and TCMR) occurred within 1 month after transplantation. The median time to rejection was 15 days in the DSA-positive subgroup (PRA^+^DSA^+^), 17 days in the DSA-negative subgroup (PRA^+^DSA^-^), and 21 days in the PRA-negative retransplant control group (PRA^-^ re-Tx).

In the presensitized group, the DSA-positive subgroup had more antibody mediated rejection, including pure AMR in two cases (8.0%) and mixed rejection in four cases (16.0%), whereas the DSA-negative subgroup had no pure AMR and only one case of mixed rejection (3.8%) (*P=*0.049). In contrast, the incidence of pure TCMR in the DSA-positive and DSA-negative subgroups was similar, 20.0% and 23.4%, respectively. **Figure 2** summarizes the time of occurrence and types of all instances of AR. The majority (88.9%) of AR occurred within 1 month and as early as 9 days after transplantation. Also, the median time of AR occurrence in the DSA-positive subgroup, DSA-negative subgroup, and control group was 15 days (IQR: 13-19 days), 17 days (IQR: 14-34 days), and 21 days (IQR: 13-27 days) after transplantation, respectively. Thus, the mean hospital stay was significantly longer in the presensitized group than in the control group (33.9 vs. 24.7 days, *P*<0.001) and in the DSA-positive subgroup than in the DSA-negative subgroup (41.8 vs. 26.3, *P*<0.001) (**Table 2**). All instances of AR in both groups were successfully cured. During the median follow-up period of 51 months in the sensitized group and 41 months in the control group, the incidence of *de novo* DSA (dnDSA) was quite low in both groups (7.8% vs. 6.5%).

In terms of other adverse events, the presensitized group had only two cases of perioperative bleeding and hematoma (3.9%), three cases of CMV viremia (5.9%), seven cases of BK virus infection (viremia and/or viruria, 13.7%), three cases of EB viremia (5.9%), three cases of pneumonia (5.9%), and nine cases of proteinuria (17.6%), with analogous groups in the control group (**Table 2**).

### Late graft function and survival

**Figure 3:**
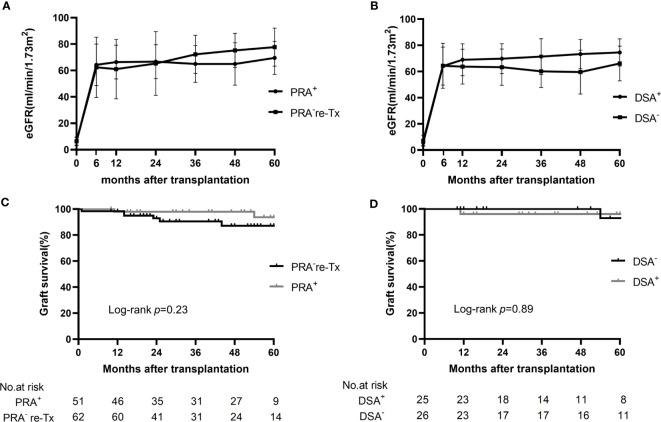
Changes in eGFR and graft survival in the control group, presensitized group, and two subgroups. **(A)** Changes in eGFR in the presensitized group (PRA^+^) and control group (PRA^-^) over 5 years of follow-up. **(B)** Changes in eGFR in the DSA-positive (DSA^+^) and DSA-negative (DSA^-^) subgroups over 5 years of follow-up. **(C)** Kaplan-Meier curves for graft survival in the presensitized group (PRA^+^) and control group (PRA^-^). **(D)** Kaplan-Meier curves for graft survival in the DSA-positive (DSA^+^) and DSA-negative (DSA^-^) subgroups.

During the 3-year follow-up period, no graft failure occurred in the presensitized group; one patient with normal allograft function died of tuberculous peritonitis in the first year, resulting in a 3-year graft and recipient survival rate of 98.0% in this group (**Table 2**, **Figure 3**).

In the control group, there were five cases of graft loss during the 3-year follow-up period: the first patient developed PNF after transplantation and died of severe donor-derived infection during the perioperative period; in the second and third recipients, graft function was gradually lost at 14 months after transplantation because of hepatitis B cirrhotic ascites and chronic allograft nephropathy (extensive interstitial fibrosis shown by renal biopsy), respectively; the fourth patient had increased serum creatinine at one month after transplantation due to TCMR, and was found to have a significantly elevated serum creatinine (910 μmol/L) 23 months after transplantation and was restarted on hemodialysis; in the case of the fifth patient, hemodialysis was restarted 25 months after transplantation because of the combined BK nephropathy, recurrent IgA nephropathy and moderately active TCMR, which was confirmed by renal biopsy. Thus, the 1-year graft and recipient survival rates were 98.4% in the control group, and the 3-year graft and recipient survival rates were 90.5% and 98.4%, respectively (**Table 2**, **Figure 3**).

The mean eGFR of the patients in the presensitized group was >60 ml/min/m^2^ at 6 months after transplantation and beyond; this result was not significantly different from that for the control group. More impressively, even the DSA-positive subgroup with the highest incidence of early acute rejection showed an allograft function comparable to that of the control group and the DSA-negative subgroup (**Figure 3**).

### Post-transplant outcome of preformed DSA

**Figure 4:**
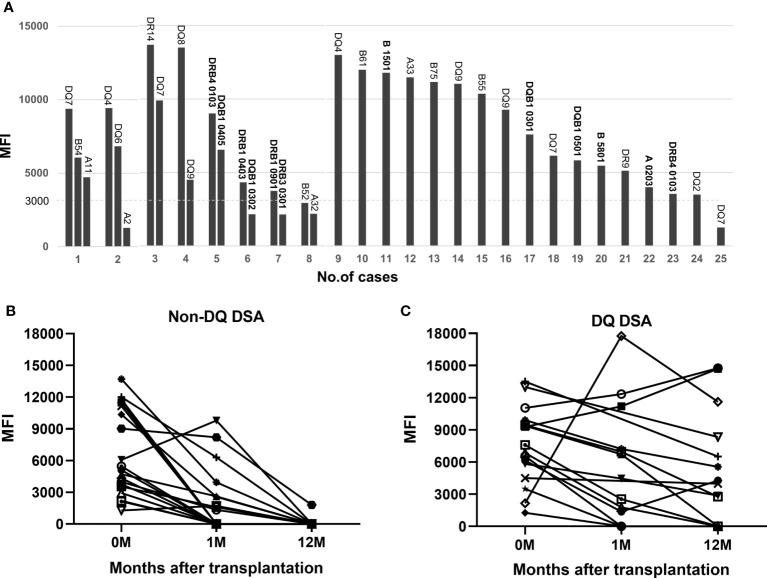
PfDSA characteristics and changes in pfDSA within 1 year after transplantation. **(A)** Types and MFI values for each pfDSA in 25 patients before transplantation. Each column represents a DSA, and each numeric label indicates the recipient number. **(B)** The evolution of non-DQ DSA; almost all of the cases (94.7%) became negative by 1 year after transplantation. Each line represents a DSA. **(C)** The evolution of the DQ-DSA; most of the cases (62.5%) were still persistently positive at 1 year after transplantation. Each line represents a DSA.

Eight patients (32.0%) in the DSA-positive subgroup had multiple pfDSAs before transplantation, including two patients with three pfDSAs and six patients with two pfDSAs; the remaining 17 patients (68.0%) had only a single pfDSA (**Figure 4**). It should be noted that 12 pfDSAs in 9 patients were identified based on second-field HLA typing results of the donor and recipient (after April 2020), and 23 pfDSAs in the remaining 16 patients were determined based on two-digital HLA typing data (before April 2020). The mean MFI value of all 35 pfDSAs was 6,999 (1,271-13,700), of which 9 were at a high level (>10,000), 13 at a medium level (5,000-10,000) and 13 at a low level (<5,000). In the pfDSA category, anti-DQ DSA was the most common, accounting for nearly half of all pfDSAs (16/35, 45.7%). The other types of DSA included anti-A DSA (5/35, 14.3%), anti-B DSA (7/35, 20%), and anti-DR DSA (7/35, 20%) (**Figure 4A**).

Six patients in the DSA-positive subgroup received PP/IVIG treatment early after renal transplantation because of biopsy-proved AMR or mixed rejection. Among the six patients, four had their AMR reversed after 3-5 sessions of PP/IVIG treatment, and one patient had the AMR reversed after 15 sessions of PP/IVIG treatment; one patient received temporary hemodialysis for 2 weeks because of AMR, and the graft function gradually returned to normal after 25 sessions of PP/IVIG in combination with low-dose splenic irradiation (10 times, 50 cGy each time). In addition, there were five other patients in the DSA-positive subgroup who received three or four sessions of PP/IVIG treatment for reasons such as maintaining a relatively high level of DSA after transplantation or having a mild rebound of DSA, in some cases combined with a slow decrease in serum creatinine.

At 1 month after transplantation, more than half (52.6%) of the pfDSAs against A, B, and DR antigens became negative, and almost all of them (94.7%) became negative by 1 year after transplantation (**Figure 4B**). In contrast, only 3 of the 16 (18.8%) anti-DQ pfDSAs disappeared at 1 month after transplantation and 10 (62.5%) were still positive 1 year after surgery (**Figure 4C**).

## Discussion

Pretransplant desensitization therapy is not very feasible for presensitized patients who are waiting for DD kidney transplantation because of this approach’s unproductive or only temporary effects (4, 16, 17). Although IdeS, an IgG endopeptidase, has been reported to be potential as a new method of desensitization due to its ability to rapidly reduce or eliminate DSA (18, 19), it is currently not available for widespread use. Therefore, how to successfully perform DD kidney transplantation in HLA-presensitized patients without prior desensitization is an important clinical issue.

Based on the previous clinical practice (20), we have emphasized the importance of donor-recipient HLA matching and perioperative treatment protocol. In the screening of immunologically suitable donors for presensitized renal transplantation, donor-recipient HLA full matching, or 0 mismatch, is the most ideal situation, followed by complete avoidance of the HLA loci targeted by preformed alloantibodies in the recipients (21). However, in the case of complete avoidance, historical HLA sensitization may also result in the rapid production of induced alloantibodies in the early period after transplantation as a result of memory immune responses, leading to acute AMR (22). Therefore, to reduce the risk of induced DSA generation, we have emphasized the importance of minimizing the number of donor-recipient HLA mismatches.

In addition, a “moderate but not excessive” perioperative immunosuppressive regimen is essential for presensitized DD kidney transplantation. The major modification in our protocol was the use of a relatively small dose of IVIG daily for 2 weeks after transplantation. Since the basic immunological requirement prior to transplantation is to be CDC negative, significant humoral injury does not occur immediately after transplantation even in the presence of DSA. At this point, the greatest risk of AMR comes from the rebound of preformed DSA or the production of induced DSA as the result of an anamnestic response by memory B cells (23–25). Therefore, we expected that the administration of daily low-dose IVIG would inhibit DSA production and significantly reduce the risk of AMR during the high-risk period, the first 2 weeks after transplantation. In the PRA^+^DSA^-^ subgroup, considering that some patients may have a certain DSA that disappeared on its own without being detected during the waiting period, and these patients are at risk for induced DSA production after transplantation, we still gave this subgroup of patients a perioperative immunosuppressive regimen similar to that in the DSA-positive subgroup.

Furthermore, we believe that selecting donor kidneys of good quality is also important for presensitized kidney transplantation; as such kidneys can effectively reduce the risk of DGF. In the absence of DGF, it is possible to closely observe the transplant patient’s urine output and recovery of renal allograft function, which can be very helpful for the early detection and diagnosis of acute AMR.

Using the principle of immunological screening of donors and the modified perioperative treatment regimen, we performed kidney transplantation in 51 presensitized kidney transplant recipients (about half DSA-positive and half DSA-negative) and achieved excellent early and intermediate-term survival results. The overall incidence of AR and AMR in the presensitized group was significantly higher than that in the non-sensitized retransplant control group. Moreover, the incidence of AMR in the DSA-positive subgroup was significantly higher than that in the DSA-negative presensitized subgroup, but the incidence of TCMR was similar between the two subgroups. These results indicate that in terms of the risk of acute rejection, the DSA-positive subgroup is at the highest risk, followed by the DSA-negative presensitized subgroup, and the group at lowest risk is the non-sensitized retransplant group. Thus, it is necessary to pay attention to the stratification of risk when implementing these types of kidney transplantation.

To date, there is no standard treatment protocol for DD kidney transplantation across DSA barriers. Tsapepas et al. reported the results of kidney transplantation in 62 patients with preformed DSA. Without desensitization therapy, the incidence of AR and AMR were 54.8% and 32.3%, respectively, and the graft survival rate was 82.1% at a median follow-up of 14.4 months (26). Schwaiger et al. evaluated transplant outcomes in 101 DSA-positive DD kidney transplant recipients who were treated with a single pretransplant immunoadsorption (IA), followed by anti-lymphocyte antibody and serial post-transplant IA. The incidence of AMR was 33%, and the 1-year and 3-year graft survival rates were 84% and 73%, respectively (27). Subsequently, Amrouche et al. reported long-term outcomes of kidney transplantation in 95 patients with high levels (MFI>3,000) of preformed DSA and a CDC negative crossmatch, most of whom (94.5%) received DD kidneys. In addition to induction therapy with high-dose thymoglobulin (1.5 mg/kg/day, for 5-10 days), a post-transplant desensitization protocol with high-dose IVIG (2 g/kg, every 3 weeks for 4 courses), plasma exchanges (5-10 times) and rituximab (1-2 times) were given from day 0. Although the incidence of AMR was still high (33%), the intensified post-transplant immunosuppressive therapy resulted in better graft survival than in the previous two studies. The 1-, 3-, 5-, and 7-year death-censored graft survival rates were 98, 91, 86 and 78%, respectively, but the incidence of infection appeared to be higher during follow-up (223 infections in total) (5).

In the present study, 25 DSA-positive DD kidney transplant recipients who received a single preoperative PP/IVIG, intraoperative rituximab (200 mg), low-dose thymoglobulin induction (total 150 mg), and daily low-dose IVIG (10-20g) within 2 weeks after transplantation achieved good early and intermediate-term survival outcomes with low infection rates. The incidence of AMR (including mixed rejection) was lower (24.0%) than in the previously mentioned studies, and the 1-year and 3-year death-censored graft survival rates were 98%. It should be emphasized that the overall DSA level of the patients in this group was also high, with 23 cases (92.0%) having an MFI > 3,000, 9 having an MFI > 10,000, and 8 having multiple DSAs. The excellent clinical results achieved in this subgroup of patients suggest that our perioperative immunosuppression regimen may be a more advantageous regimen than those mentioned earlier.

To our knowledge, there have been no reports of daily low-dose IVIG treatment in the early postoperative period of presensitized renal transplantation. In the present study, no AMR was found within the first week after renal transplantation in the presensitized recipients. The majority of AMR episodes occurred in the third week after transplantation, when IVIG had been discontinued. Moreover, the incidence of AMR was relatively low, and most of the cases could be easily reversed by treatment. These results suggest that daily low-dose IVIG therapy may play an important role in preventing, delaying, or alleviating AMR in presensitized renal transplantation. In addition, when compared to the high postoperative dose of IVIG (2g/kg) reported in the literature (5), daily administration of a dose of 10g or 20g in our study was more easily tolerated by patients. The low incidence of post-transplant infection in our study may also be associated with the use of IVIG for 2 weeks.

In this study, some patients had high preoperative MFI values for DSA but negative flow-CDC, which may be related to the IgG subtype of DSA. It has been reported that non-consensus CDC-XM was always in presence of HLA IgG DSA and that laboratories may be struggling to interpret the low sensitive CDC-XM results, where highly sensitive solid phase multi-antigen or single antigen assay shows the presence of HLA IgG DSA in serum (28). The extent of complement activation depends on the isotype of anti-HLA antibodies. For example, the IgG3 subclass of donor-specific anti-HLA antibodies has been reported as a more potent complement-fixing IgG subclass (29, 30). Therefore, if the subtype of DSA is an IgG subclass with weak complement activation, the Flow-CDC result may be negative.

We are aware of some shortcomings and noteworthy aspects of this study: 1) the number of cases reported in this study is limited and the follow-up time is not long enough; 2) not all pfDSAs were identified based on second-field HLA typing results of the donor and recipient, which may result in a few pfDSAs we had shown not being true DSAs; 3) Since DSA-negative presensitized recipients have a lower risk of AMR after kidney transplantation, the need for us to follow a similar perioperative treatment regimen in this subgroup as in the DSA-positive subgroup needs to be further validated.

In conclusion, we have used a novel IVIG-based perioperative treatment protocol for DD kidney transplantation in presensitized patients and achieved excellent intermediate-term graft and recipient survival results. In addition, our study provides evidence that selected DSA-positive patients can be successfully transplanted across the HLA barrier. Given that this is a single-center experience report, the effect of our strategy needs to be verified by larger, multi-center studies in the future.

## Data availability statement

The original contributions presented in the study are included in the article/supplementary material. Further inquiries can be directed to the corresponding authors.

## Ethics statement

The studies involving human participants were reviewed and approved by the studies involving human participants were reviewed and approved by Medical Ethics Committee, Tongji Hospital of Tongji Medical College, Huazhong University of Science and Technology. Written informed consent for participation was not required for this study in accordance with the national legislation and the institutional requirements.

## Author contributions

ZG participated in performance of the study, data acquisition and analysis, and writing of the manuscript. DZ and GZ participated in transplant surgery. RS participated in data collection. LW and SL participated in typing and antibody detection. LZ participated in study implementation, patient management, data analysis and manuscript editing. GC had substantial contributions to the conception and design of the work and interpretation of data. In addition, he performed most surgeries and critically revised the article. All authors contributed to the article and approved the submitted version.
